# New Appliances and Things Medical

**Published:** 1895-07-20

**Authors:** 


### NEW APPLIANCES AND THINGS MEDICAL.

[We shall be glad to receive, at our Office, 428, Strand, London, W.C., from the manufacturers, specimens of all new preparations and appliances which may be brought out from time to time.]

### THE "EVER READY CADDY."

(Ferris and Co., Bristol.)

The inventors and manufacturers of the above have sent us an extremely neat mahogany cabinet containing twelve of the caddies. The first thing one does after looking it over is to say, "Why wasn't this made before?" It fills so obvious a place in the surgery and consulting-room, and saves such a lot of trouble by finding a place for dressings, strappings, and bandages, and keeps them altogether in one corner or on the mantelshelf, "ever ready" to the hand when wanted, and free from germ-laden dust or the contact of the septic, prying fingers of the casual visitor to the doctor's sanctum. The caddy, made in various sizes, is an oblong tin box with a lid, something like a Huntley and Palmer's small biscuit-box. Through the centre of the caddy runs a rod, on which the cylinder—round which the dressings, plasters, &c., are wound—moves. To fill the caddy all that it is necessary to do is to pull out the shutter at one end, draw out the rod, place the dressing (already wound on its cylinder) in the box, pass the rod through it, press the rod home, drop in the shutter, and the dressing is ready to be unwound and cut off in suitable lengths for the purpose for which it may be required. The cabinet sent to us contains twelve caddies, which hold the following selection from Messrs. Ferris and Co.'s extensive list: Six yards emplast. adhesive (Bristol strapping on pure calico), self-adhesive antiseptic rubber plaster on spools (seven of different widths), three yards emplast belladon on swansdown, six yards universal protective, half-a-pound hospital lint, twelve yards full-width sublimate gauze, one roll carbolised wool, twelve yards full width iodoform gauze, a packet of Gamgee tissue, a roll of absorbent wool, half-a-pound boracic lint, and half-a-pound carbolic lint. With a selection like this the busy practitioner has always to hand clean dressings suitable to the varied cases which crop up from day to day. These dressings the manufacturers keep ready rolled on the cylinders, so that when the caddy is empty it only requires the momentary trouble of filling, as above described, to start afresh. We congratulate the inventors on filling a real want, and also on producing a contrivance which, although of a simple kind, will yet help considerably in keeping the dressings aseptic by finding a caddy for everything and everything in a caddy, and thus avoiding the risks of contamination by carelessness.

